# Diseases of the Heart; Their Diagnosis and Treatment

**Published:** 1902-09

**Authors:** 


DISEASES OF THE HEART; THEIR DIAGNOSIS AND TREATMENT. By Albert Abrams, A. M., M. D., San Francisco, Consulting Physician for Diseases of the Chest, Mt. Zion Hospital and the French Hospital. Illustrated. Pages, 172. Price, $1 net.

In this book the author discusses the subject of diseases of the heart entirely from a practical aspect. His most noteworthy researches in methods of diagnosis are here recorded for the first time in collected form, and the latest and most practical methods of treatment given in detail. T. J. B.

